# Effect of β-Cyclodextrin on the Mixed Micellization of Triton X-100 and Brij S20: A Thermodynamic Study

**DOI:** 10.3390/ijms27146284

**Published:** 2026-07-15

**Authors:** Kosta Popović, Zita Farkaš Agatić, Ana Pilipović, Mihalj Poša

**Affiliations:** Department of Pharmacy, Faculty of Medicine, University of Novi Sad, Hajduk Veljkova 3, 21000 Novi Sad, Serbia; kosta.popovic@mf.uns.ac.rs (K.P.); zita.farkas@mf.uns.ac.rs (Z.F.A.); mihalj.posa@mf.uns.ac.rs (M.P.)

**Keywords:** β-cyclodextrin, Triton X-100, Brij S20, mixed micelles, inclusion complexes, regular solution theory, thermodynamics, micellization

## Abstract

The micellization behavior of binary mixtures of the nonionic surfactants Triton X-100 and Brij S20 was investigated in aqueous solution in the absence and presence of β-cyclodextrin (βCD) over the temperature range 283.15–318.15 K. Critical micelle concentrations (*CMC*) were determined and analyzed using regular solution theory to evaluate mixed micelle composition, interaction parameters, activity coefficients, and excess Gibbs free energies. The Triton X-100/Brij S20 system exhibited pronounced synergistic interactions, reflected by negative interaction parameters, reduced *CMC* values, and negative excess Gibbs free energies over the entire composition range. The addition of βCD systematically increased *CMC* values and reduced the magnitude of the interaction parameters, indicating that inclusion complex formation competes with micellization and weakens surfactant–surfactant interactions. Corrected micellar compositions revealed significant redistribution of surfactants between the aqueous phase and mixed micelles due to host–guest complexation. Thermodynamic analysis demonstrated spontaneous formation of βCD inclusion complexes with both surfactants, characterized by negative Gibbs free energy, enthalpy, and entropy changes. Triton X-100 exhibited a higher affinity toward βCD than Brij S20. The results demonstrate that coupled equilibria between micellization and inclusion complex formation govern the behavior of the system and suggest that conformational contributions should be considered when interpreting interaction parameters in cyclodextrin–surfactant systems.

## 1. Introduction

The study of interactions between cyclodextrins and surfactants in aqueous solution involves monitoring and analyzing multiple concurrent processes that occur simultaneously and influence one another. Cyclodextrins are well-characterized cyclic oligosaccharides composed of glucopyranose units linked by α-1,4-glycosidic bonds, obtained through enzymatic starch degradation [1,2]. The three most common forms consisting of six, seven and eight D- glucopyranose units—α-cyclodextrin (αCD), β-cyclodextrin (βCD), and γ-cyclodextrin (γCD)—serve as funda-mental building blocks for the synthesis of numerous derivatives with enhanced physicochemical properties [3]. Due to their unique structure, cyclodextrins exhibit the ability to form host–guest inclusion complexes with lipophilic substances [4,5,6,7,8].

In the supramolecular complexes they form, cyclodextrins act as host molecules whose hydrophobic cavities accommodate hydrophobic guest molecules or their nonpolar moieties. A key driving force in this process is the hydrophobic effect: classical, primarily entropic in origin and associated with the displacement of energetically unfavourable water molecules from the cyclodextrin cavity upon guest inclusion [9,10], and the non-classical hydrophobic effect that involves the structuring of water molecules at the interface of the hydrophobic domains of both the host and the guest [10,11]. In addition, inclusion complexation is influenced by hydrogen bonding and electro-static interactions [12].

Surfactants are surface-active agents that adsorb at the interface between two phases in a solution, where they exert their characteristic effects. Due to their amphiphilic structure, surfactants exhibit a strong tendency to self-assemble in aqueous solutions. When present at concentrations above their critical micelle concentration (CMC), they aggregate to form structures known as micelles. If the aqueous solution contains more than one surfactant particle, then mixed micelles form. Mixed micelles also form at a CMC corresponding to the surfactant mixture’s total concentration, where the surfactant molar ratio is defined [13,14,15]. Owing to their structural characteristics, surfactant molecules can also interact with cyclodextrins in aqueous media, leading to the formation of inclusion complexes in which the hydrophobic segments of the surfactant are partially or fully accommodated within the cyclodextrin cavity.

**Figure 1:**
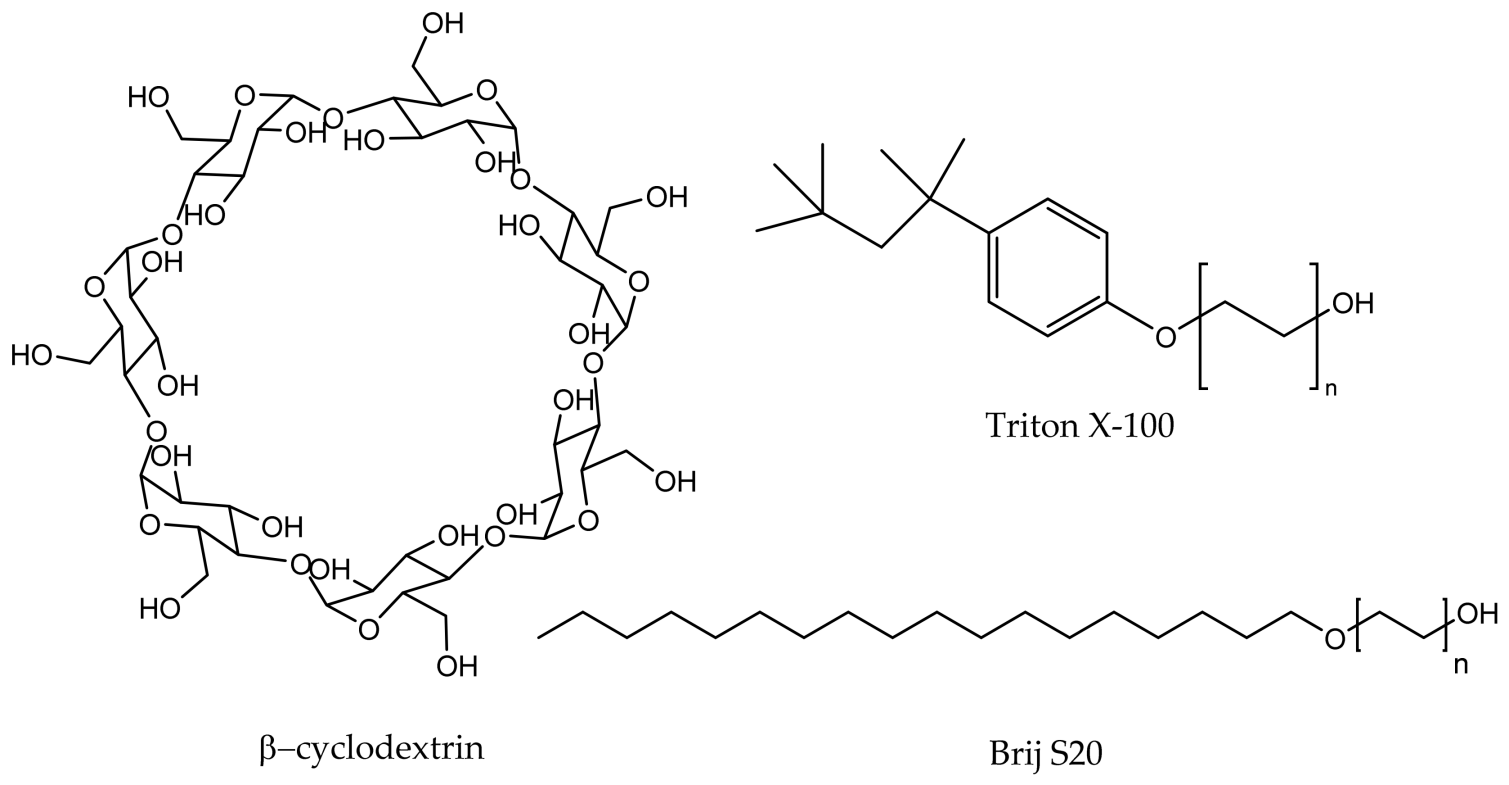
Structures of examined molecules: β- cyclodetrin, Brij S20 and Triton X-100.

In this study, interactions in aqueous solution between β-cyclodextrin, a well-characterized cyclodextrin composed of seven glucopyranose units and two nonionic surfactants, Brij S20 and Triton X-100 (Figure 1), were investigated. This was achieved through the experimental determination of their critical micelle concentrations (CMC) and the calculation of thermodynamic parameters that describe the processes occurring within this system.

Nonionic surfactants are widely employed in numerous industrial applications due to their versatile properties. Brij surfactants represent a typical class of nonionic surfactants, characterized by a hydrophilic head group and a hydrophobic tail. In the case of Brij S20 (BS20), the hydrophilic portion consists of a polyoxyethylene (PEO) chain containing, on average, 20 oxyethylene (OE) units, attached to a hydrophobic stearyl chain.

Triton X-100 (TX100) is also a nonionic surfactant; however, it differs structurally from conventional representatives of this group: it contains an alkyl–aryl (octylphenyl) hydrophobic moiety. Owing to this distinct structure, Triton X-100 is extensively used in biochemical research and in various pharmaceutical applications [16,17,18].

**Scheme 1:**
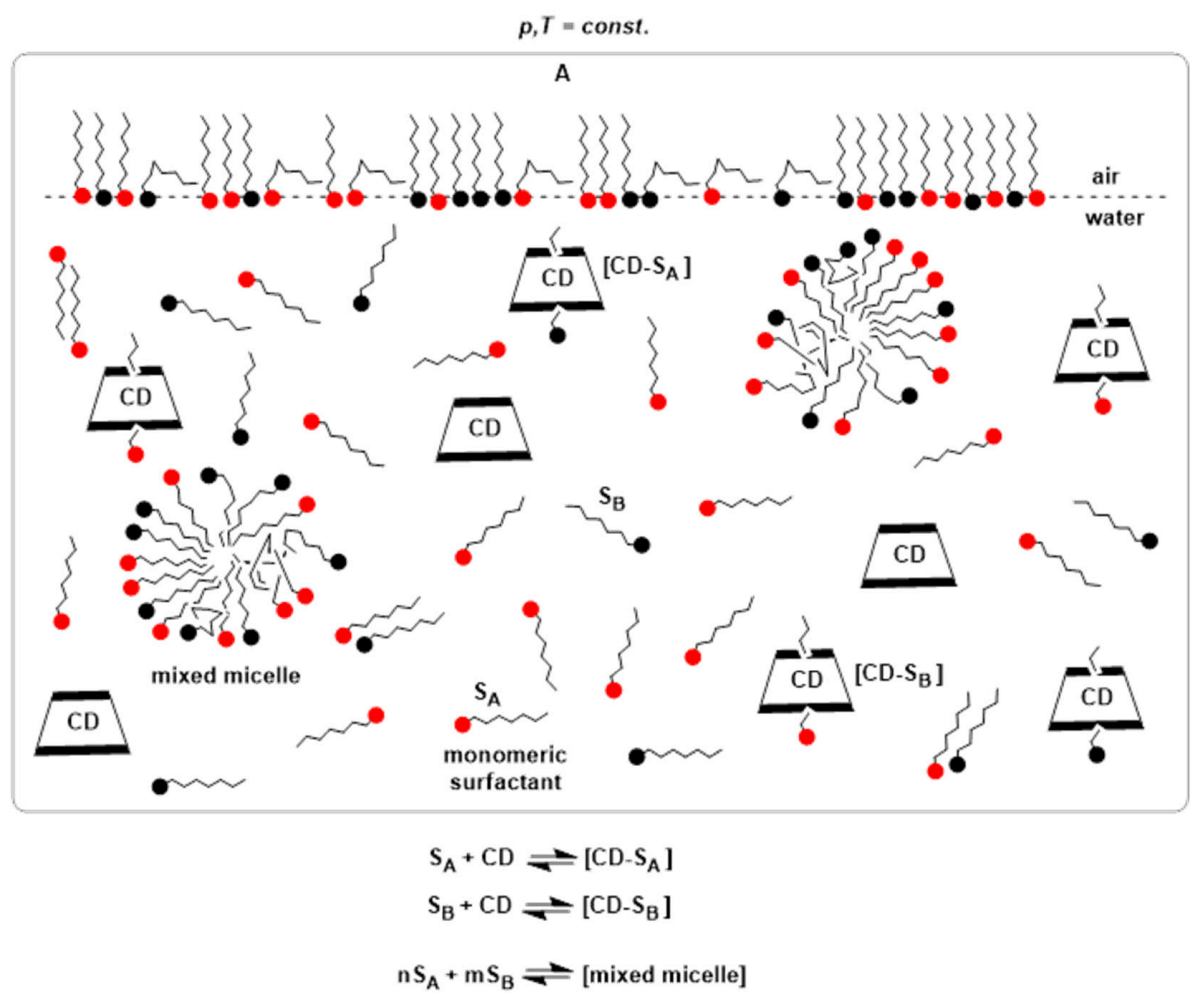
Equilibrium state (A) in an aqueous solution containing surfactant monomers A and B (SA and SB), binary mixed micelles, free cyclodextrin (CD), and inclusion complexes (CD–SA and CD–SB), at a constant total surfactant concentration (above the critical micelle concentration of the binary surfactant mixture) and a constant total cyclodextrin concentration. The system is governed by the competitive equilibria between mixed micelle formation and inclusion complex formation.

The aim of this study was to investigate the influence of β-cyclodextrin on the micellization behavior of binary mixtures of the nonionic surfactants Triton X-100 and Brij S20 in aqueous solution over the temperature range 273.15–318.15 K. Particular attention was devoted to the competition between mixed micelle formation and cyclodextrin–surfactant inclusion complexation. The Scheme 1 highlights the competition between micellization and host–guest complex formation of the examined molecules. Critical micelle concentrations were experimentally determined, and the obtained data were analyzed using Regular Solution Theory in order to evaluate mixed micelle composition, interaction parameters, activity coefficients, and excess Gibbs free energies. Furthermore, equilibrium constants and thermodynamic parameters associated with β-cyclodextrin inclusion complex formation were determined to elucidate the molecular interactions governing the coupled equilibria between micellization and host–guest complexation.

## 2. Results

### 2.1. Micellization Behavior of Triton X-100/Brij S20 Mixed Surfactants

Table 1 summarizes the experimentally determined critical micelle concentrations (*CMC*) of mixed micelles composed of Triton X-100 and Brij S20 at various molar ratios (*α* denotes the mole fraction of Triton X-100) over the temperature range 273.15–318.15 K, with 5 K increments. In addition, the corresponding thermodynamic parameters were evaluated. The ideal critical micelle concentrations (*CMCid*) were calculated using Clint’s equation [1]. Interactions in ideal binary micelles are identical to interactions (and or conformational states of surfactants) that exist in monocomponent micelles of surfactant building units. Furthermore, the mole fractions of both cosurfactants in the micelle (*x_i_* and *x_j_*), the interaction parameter (*β*), the activity coefficients (*f_i_* and *f_j_*), and the molar excess Gibbs free energy (*G^e^*) were determined within the framework of regular solution theory (RST) using the Rubingh–Holland protocol [19,20,21,22].

### 2.2. Competition Between Micellization and Inclusion Complex Formation

In aqueous surfactant/cyclodextrin systems, micellization and inclusion complex formation occur concurrently. An equilibrium is established among free surfactant monomers, micellar aggregates of various aggregation numbers, and surfactant molecules incorporated into cyclodextrin inclusion complexes. Since the formation of cyclodextrin–surfactant inclusion complexes decrease the concentration of free surfactant molecules in solution, a higher total surfactant concentration is required for the free surfactant concentration to attain the critical micelle concentration (*CMC*) characteristic of the cyclodextrin-free system.

According to the theory of detailed balance, the equilibrium constant for micelle formation in the presence of cyclodextrin remains identical to that in its absence, provided that cyclodextrin–surfactant inclusion complexes do not participate significantly in interfacial adsorption [23]. Although inclusion complex formation reduces the concentration of free surfactant molecules available for micellization, the critical micelle concentration of the free surfactant monomer is expected to remain essentially unchanged (*CMC = const.*) [23]. In practice, however, the surface activity of cyclodextrin–surfactant inclusion complexes may depend on their structure. When a portion of the hydrophobic chain extends outside the cyclodextrin cavity, the complex may retain some ability to adsorb at the air/water interface, although the large cyclodextrin headgroup generally prevents efficient micelle formation.

However, the experimentally determined critical micelle concentration in the presence of cyclodextrin (${CMC}_{EXP}$) is higher than the intrinsic *CMC* (i.e., *CMC* value of the surfactant in aqueous solution without cyclodextrin) value by an amount corresponding to the concentration of surfactant monomers incorporated into the cyclodextrin cavity ($C_{MONOMERIC}$):

(1) $${CMC}_{EXP}=CMC+C_{MONOMERIC\;}$$

This relationship applies in the case of a 1:1 inclusion complex. The equilibrium concentration of the cyclodextrin–surfactant inclusion complex with 1:1 stoichiometry, $C_{CD-S(eq)}$, in aqueous solution is therefore:

(2) $$C_{CD-S\left(aq\right)}={CMC}_{EXP}-CMC=C_{MONOMERIC}$$

The equilibrium concentration of free cyclodextrin is given by the difference between the total cyclodextrin concentration, $C_{CD}^{T}$, and the equilibrium concentration of the inclusion complex, $C_{CD-S(eq)}$:$\;C_{CD}^{T}-C_{CD-S(eq)}$. Accordingly, the equilibrium constant for the formation of a 1:1 cyclodextrin–surfactant inclusion complex can be expressed as follows [23,24]:

(3) $$K=\frac{C_{CD-S(eq)}}{CMC\left(C_{CD}^{T}-C_{CD-S(eq)}\right)}=\frac{{CMC}_{EXP}-CMC}{{CMC(C_{CD}^{T}-C(MC}_{EXP}-CMC))}$$

The equilibrium constant for the formation of the 1:1 β-cyclodextrin–surfactant inclusion complex was determined using the graphical method proposed by Poša [23,24]. According to Equation (3), the experimental data were linearized by plotting the dependence of ${(CMC}_{EXP}-CMC)$ (y-axis) against $CMC\left(C_{CD}^{T}-{(CMC}_{EXP}-CMC\right)$ (x-axis).

For each temperature and cyclodextrin concentration, a linear least-squares regression was applied using Microsoft Excel. The slope of the resulting linear fit corresponds to the equilibrium constant K. The quality of the linear fit was evaluated using the coefficient of determination (R^2^). The uncertainty in K was estimated from the standard error of the slope obtained from the regression analysis and propagated to the thermodynamic parameters (ΔG, ΔH and ΔS).

In previous studies, the inclusion complex between β-cyclodextrin and Triton X-100 has most commonly been described by a 1:1 host–guest stoichiometry [25,26]. Banik et al. confirmed a well-defined 1:1 binding mode, suggesting that the hydrophobic tail of the surfactant is selectively included within the β-cyclodextrin cavity, while the hydrophilic poly(ethylene oxide) chain remains exposed to the aqueous phase [25]. However, other studies have also reported the coexistence of both 1:1 and 2:1 (βCD: TX-100) complexes, depending on the experimental conditions, surfactant concentration, and the experimental technique employed [27,28].

Unlike Triton X-100, the stoichiometry of the β-cyclodextrin–Brij S20 inclusion complex has not been clearly established. The thermodynamics of inclusion complex formation between β-cyclodextrin and nonionic surfactants (Brij series: Brij 56, 58, 76 i 78) were investigated using isothermal titration calorimetry (ITC) [5]. The results indicate that β-cyclodextrin can interact with both the hydrophobic alkyl chain and the hydrophilic ethoxylated segment of the surfactant molecules. This behavior arises from the lack of a specific binding site that would restrict the position of β-cyclodextrin along the surfactant structure. As a result, the inclusion process is dynamic and can occur at multiple regions of the surfactant molecule. Therefore, multiple binding modes may coexist rather than a single well-defined host–guest geometry. Nevertheless, in the present work the thermodynamic analysis was performed assuming an apparent 1:1 host–guest stoichiometry, which represents the basis of the applied equilibrium model (Equation (3)). This assumption provides a consistent framework for comparing the inclusion behavior of Triton X-100 and Brij S20, while acknowledging that more complex binding equilibria may exist.

### 2.3. Effect of β-Cyclodextrin on the Micellization of Triton X-100 and Brij S20

In Table 2, Table 3 and Table 4 are presented experimentally obtained values of critical micelle concentrations (*CMC*) of mixed micelles composed of Triton X-100 and Brij S20 at various molar ratios (*α* denotes the mole fraction of Triton X-100) over the temperature range *T* = (273.15–318.15) K, with 5 K increments in the presence of 0.5 mmol/dm^3^, 1 mmol/ dm^3^ and 2 mmol/dm^3^ β-cyclodextrin. In addition, the corresponding thermodynamic parameters were evaluat-ed: The ideal critical micelle concentrations (*CMCid*), the mole fractions of both cosurfactants in the micelle (*x_i_* and *x_j_*), the interaction parameter (β_ij_), the activity coefficients (*f_i_* and *f_j_*), and the excess Gibbs free energy (*G^e^*) were determined within the framework of regular solution theory (RST) using the Rubingh–Holland method [21,22]. However, as the two surfactants have different affinities towards the formation of the inclusion complex, then, formally after the formation of the inclusion complex, the ratio (mole fraction) of the surfactant in their virtual binary mixture (from which a mixed micelle is formally formed) changes in relation to their original binary mixture where the mole fraction levels are defined by $\alpha_{i}$ values. Therefore, a modified RST model that takes into ac-count changes in alpha values is necessary. The composition of the mixed micelles, according to the modified RST model, *x_i_* = *x_i_*(*K*) and *x_j_* = *x_j_*(*K*), was determined using Equations (4) and (5) [21,22], applying the interaction parameter (*β*) obtained for the corresponding binary surfactant system in the absence of cyclodextrin.

(4) $$\frac{\alpha_{i}{CMC}_{mix}^{CD}-{CMC}_{i}x_{i}(\exp\left[\beta {\left(1-x_{i}\right)}^{2}\right]}{K\left[CD-i\right]{CMC}_{i}x_{i}\exp\left[\beta {\left(1-x_{i}\right)}^{2}\right]}=\frac{\alpha_{j}{CMC}_{mix}^{CD}-{CMC}_{j}x_{j}\exp\left[\beta {\left(1-x_{j}\right)}^{2}\right]}{K\left[CD-j\right]{CMC}_{j}x_{j}\exp\left[\beta {\left(1-x_{j}\right)}^{2}\right]}$$

(5) $$x_{i}+x_{j}=1$$

Namely, according to Guggenheim’s original theory of regular mixtures, the interaction coefficient does not depend on the $\alpha_{i}$ value [29]. At low and at high $\alpha_{i}$ values, the interaction parameter may deviate significantly due to the error in determining the critical micellar concentrations of binary surfactant mixtures dominated by one sur-factant [30]. As according to Guggenheim’s RST, the interaction parameter does not depend on the mole fractions of the components of the binary mixture, therefore the goal of applying the original RST is to check the influence of the presence of cyclodextrin in the aqueous solution of surfactants on the interaction parameter.

Based on the calculated mole fractions in the micellar pseudophase and the corresponding interaction parameters (*β_ij_*), the concentration of inclusion complexes and the distribution of surfactants within the system were determined.

The critical micelle concentration (*CMC*) values obtained by tensiometric measurements are in good agreement with, and confirm, the *CMC* values determined by spectrofluorimetric analysis using pyrene as a probe molecule, as presented in Table 1, Table 2, Table 3 and Table 4 and are presented in the Appendix A.

### 2.4. Thermodynamics of β-Cyclodextrin–Triton X-100 Inclusion Complex Formation

Table 5 presents the calculated standard equilibrium constants for the formation of inclusion complexes between Triton X-100 and different concentrations of β-cyclodextrin (0, 0.5, 1, and 2 mmol/dm^3^), denoted as *K*[CD–S], obtained using a graphical method. In this approach, the equilibrium constant is determined from the slope of the linear dependence between $∆CMC=CMC\left(CD\right)-CMC(S)$ and the corresponding concentration term $CMC\left(S\right)*C_{CD}^{T}$), based on the measured values of $C_{CD}^{T}$, $CMC\left(S\right),CMC\left(CD\right).$ Table 5 also includes the calculated thermodynamic parameters. For the calculation of the thermodynamic parameters, the dimensionless value of the standard equilibrium constant was used. This approach ensures that the calculated Gibbs free energy changes are expressed relative to the standard state, as required by the thermodynamic relationship ∆*G*^0^ = −*RT* ln*K*^0^. In addition, the enthalpy and entropy changes were determined from the temperature dependence of Δ*G* using the linear form:

(6) $$\frac{∆G}{T}=\frac{∆H}{R}\frac{1}{T}-\frac{∆S}{R}$$

where *R* = 8.314 J·mol^−1^·K^−1^. From the slope and intercept of the Δ*G* versus 1/*T* plot, Δ*H* and Δ*S* were obtained as Δ*H* = slope × *R* and Δ*S* = −intercept × *R*, respectively.

### 2.5. Thermodynamics of β-Cyclodextrin–Brij S20 Inclusion Complex Formation

Table 6 presents the calculated standard equilibrium constants for the formation of inclusion complexes between Brij S20 and β-cyclodextrin, denoted as K[CD–S], together with the corresponding thermodynamic parameters Δ*G*, Δ*H* and Δ*S*.

## 3. Discussion

### 3.1. Micellization Behavior of Triton X-100/Brij S20 Mixed Surfactants

Related to our previous preliminar work [31], in which the same surfactant system was investigated (without prior purification) using the same experimental methodology, prior to use in the present study, Brij S20 was purified by recrystallization from ethanol, ensuring a higher degree of sample purity.

Although some differences in the numerical values of the calculated parameters were observed, the overall behavior of the system remained unchanged. In particular, the interaction parameter (*β_ij_*) exhibited the same concentration and temperature-dependent trends in both studies, confirming the consistency and reproducibility of the observed intermolecular interactions. Therefore, the conclusions drawn from the preliminary investigation are fully supported by the more extensive experimental dataset presented in this work.

The TX-100/Brij S20 mixed system (Table 1) shows stronger synergistic interactions and greater deviation from ideal behavior compared to the previously reported results. The consistently negative *β_i_*_j_ and *G^E^* values indicate favorable intermolecular interactions and spontaneous mixed micellization over the entire temperature range. Furthermore, the smoother temperature dependence observed in the present study suggests enhanced thermodynamic consistency and compatibility between the two nonionic surfactants.

The experimental *CMC* values of mixed Triton X-100/Brij S20 micelles show a nonlinear dependence on mole fraction (*α*). At most temperatures, the *CMC* values are lower than those of the pure components, particularly in the intermediate composition range (*α* = 0.2–0.6), indicating synergistic interactions and facilitated micelle formation. The experimental CMC values are generally lower than the ideal values (*CMCid*), confirming favorable intermolecular interactions and deviation from ideal behavior.

With increasing temperature, the *CMC* values of both pure and mixed systems generally increase, suggesting less favorable micellization at higher temperatures. However, mixed systems exhibit minimum *CMC* values at intermediate compositions, indicating optimized molecular packing within the micelles. The interaction parameter βij is negative over the entire temperature range, confirming synergistic interactions between Triton X-100 and Brij S20. Comparison of the mole fraction of Triton X-100 in the mixed micelle (*x_i_*) with its overall mole fraction in the bulk solution, *α*(TX100), reveals that *x_i_* is generally lower than *α*(TX100) throughout the investigated composition range. This indicates that the mixed micelles are enriched in Brij S20, the surfactant exhibiting the lower CMC value. Such behavior is expected since Brij S20 possesses a greater affinity for the micellar pseudophase and therefore preferentially participates in micelle formation. Consequently, the composition of the mixed micelles deviates from the bulk composition and becomes richer in Brij S20 than predicted for an ideal mixture.

Furthermore, the activity coefficients of both surfactants are lower than unity over the entire composition range investigated. Values of *f_i_* and *f_j_* below 1 indicate that both Triton X-100 and Brij S20 are thermodynamically more stable in the real mixed micellar pseudophase than in an ideal mixed micelle. This observation is consistent with the negative values of the interaction parameter *β_ij_* and the excess Gibbs free energy of mixing (*G^E^*), confirming attractive interactions between the two nonionic surfactants and synergistic mixed micelle formation.

The negative values of *G^E^* (Table 1) indicate that the Gibbs free energy of the real mixed micellar pseudophase is lower than that of the corresponding ideal mixed micellar pseudophase, reflecting favorable interactions between Triton X-100 and Brij S20.

Overall, the Triton X-100/Brij S20 system exhibits pronounced nonideal mixing with strong synergistic interactions, leading to reduced CMC values and enhanced stability of mixed micelles, while elevated temperature weakens these interactions and decreases micellar stability.

### 3.2. Competition Between Micellization and Inclusion Complex Formation; Effect of β-Cyclodextrin on the Micellization of Triton X-100 and Brij S20

The obtained results (Table 2, Table 3 and Table 4) clearly demonstrate that the presence of β-cyclodextrin (βCD) significantly affects the micellization behavior of Triton X-100/Brij S20 mixed systems. In the absence of βCD, the system exhibits pronounced synergistic interactions, as evidenced by strongly negative interaction parameters (*β_ij_*) and relatively low critical micelle concentration (*CMC*) values, indicating favorable mixing and efficient micelle formation.

Consistent with previous studies, β-cyclodextrin readily forms inclusion complexes with nonionic surfactants, thereby influencing their aggregation behavior. However, unlike most previous investigations, which focused on individual surfactants, the present study examines a binary nonionic surfactant system, providing new insight into the coupled equilibria between mixed micellization and β-cyclodextrin inclusion complex formation [5,32,33,34,35].

Previous calorimetric studies on β-CD/Brij systems demonstrated that β-CD can interact with both the hydrophobic alkyl chain and the polyoxyethylene segment of nonionic surfactants, resulting in dynamic inclusion behavior. Complex formation was found to be predominantly enthalpy-driven, in agreement with the present thermodynamic results [5,34].

The present results are consistent with these observations. The addition of β-CD significantly increased the experimentally determined CMC values of both Triton X-100 and Brij S20, as well as of their mixed micelles, indicating that a considerable fraction of surfactant molecules became incorporated into cyclodextrin cavities before participating in micellization. This increase became progressively larger with increasing β-CD concentration, confirming that inclusion complex formation competes directly with micelle formation.

The interaction parameter *β_ij_* remains negative in all investigated systems, indicating that attractive interactions between Triton X-100 and Brij S20 persist even in the presence of βCD. However, a clear trend toward less negative *β_ij_* values is observed with increasing βCD concentration. This reduction in magnitude of *β_ij_* confirms that βCD weakens synergistic interactions between the surfactants. In other words, the presence of βCD disrupts the optimal packing of surfactant molecules within the mixed micelles, reducing cooperativity in micelle formation.

Unlike previous studies, which focused exclusively on individual surfactants, the present work demonstrates that inclusion complex formation also modifies the composition of mixed micelles. In the absence of β-CD, the interaction parameter βij was strongly negative, indicating pronounced attractive interactions between Triton X-100 and Brij S20 and significant synergism during mixed micellization. After addition of β-CD, the absolute values of βij generally decreased, suggesting partial disruption of favorable surfactant–surfactant interactions due to selective host–guest complex formation. Nevertheless, βij remained negative throughout the investigated composition range, demonstrating that mixed micelles remained thermodynamically more stable than predicted for ideal mixtures.

Since the interaction parameter between two different surfactants in a binary mixed micellar pseudophase, if βCD is present in the aqueous phase, is significantly different from the interaction parameters in a system without βCD, it means that the Guggenheim model of regular mixtures, according to which the interaction parameter depends exclusively on the difference in the energy of intermolecular interactions between different and identical surfactants, cannot be accepted, but that conformational effects also play a role in defining the interaction parameter, i.e., the interaction parameter corresponds more closely to the Margules parameters. Namely, as Triton X-100 has a greater affinity towards the formation of an inclusion complex than Brij S20, then its mole fraction in the mixed micellar pseudophase decreases compared to the mole fraction of the same system without βCD, which means that in the micellar hydrophobic core for the hydrocarbon chain of Brij S20, more space remains so that it can narrow down different conformations, starting from the linear and antiperiplanar to the globular conformation—it increases conformational entropy that enters the interaction parameter. The interaction parameter has the characteristic of Helmholtz free energy (hydrocarbon chains of different conformations correspond to systems of different energies, i.e., at a constant temperature they form a canonical set (ensemble)). Therefore, it is necessary to include conformational effects (internal degrees of freedom) in the Guggenheim interaction parameter.

Although the regular solution theory is widely applied to describe mixed micellar systems, it is based on several simplifying assumptions. The model assumes the formation of a single homogeneous mixed micellar pseudophase with a constant aggregation number and ideal equilibrium between monomeric and micellar surfactants. In the present system, these assumptions are only approximately fulfilled because β-cyclodextrin simultaneously forms inclusion complexes with surfactant monomers, thereby introducing an additional competing equilibrium. Consequently, the experimentally determined interaction parameters should be regarded as apparent thermodynamic parameters reflecting not only surfactant–surfactant interactions within mixed micelles but also the redistribution of surfactant molecules between free monomers, mixed micelles, and β-cyclodextrin inclusion complexes. Nevertheless, the regular solution theory remains a useful framework for comparing interaction trends and evaluating the influence of β-cyclodextrin on mixed micellization under identical experimental conditions. Future development of thermodynamic models capable of simultaneously describing mixed micellization and cyclodextrin complexation would provide a more comprehensive description of these coupled equilibria.

Upon addition of βCD (0.5–2 mM), a systematic increase in *CMC* values is observed across all molar fractions and temperatures. This trend suggests that βCD reduces the effective concentration of free surfactant monomers available for micellization. This behavior can be attributed to the formation of inclusion complexes between βCD and surfactant molecules, particularly Triton X-100, which is known to strongly interact with cyclodextrins due to its aromatic hydrophobic moiety. As a consequence of this complexation, affinity for micelle formation decreases, resulting in higher *CMC* values and delayed micellization. At higher βCD concentrations (2 mM), the system approaches a regime of significantly weakened interaction, suggesting that inclusion complexation competes effectively with micellization. The mole fractions of surfactants in the micelle (xi and xj) show noticeable deviations from the overall bulk composition, confirming non-ideal mixing behavior. However, with increasing βCD concentration, these deviations become less pronounced, indicating a reduction in selective incorporation of surfactants into the micellar core.

Additionally, activity coefficients (fi and fj) tend to approach unity at higher βCD concentrations, which may appear to indicate reduced non-ideality. However, this effect is not due to ideal mixing but rather to disruption of surfactant–surfactant interactions by βCD, which introduces a competing association pathway (inclusion complex formation). The excess Gibbs free energy (*G^e^*) remains negative for all systems, confirming that micellization is thermodynamically favorable under all investigated conditions. However, a clear decrease in the magnitude of *G^e^* is observed with increasing βCD concentration. An increase in temperature generally leads to higher *CMC* values and a reduction in the magnitude of *β_ij_* and *G^e^* across all systems. This behavior suggests that elevated temperatures weaken hydrophobic interactions and reduce the stability of mixed micelles. The combined effect of temperature and βCD is synergistic in destabilizing micelle formation, leading to progressively less favorable thermodynamic conditions at higher temperatures and higher βCD concentrations.

At low or zero βCD concentration, micellization dominates, leading to strong synergistic interactions and low *CMC* values. As βCD concentration increases, inclusion complexation becomes increasingly significant, effectively reducing the availability of free surfactant monomers and disrupting micellar packing. Consequently, the system transitions from a strongly synergistic mixed micellar regime to a competition-dominated regime in which micellization is progressively hindered.

The calculated micellar compositions *x_i_*(*K*) and *x_j_*(*K*) reveal that the presence of β-cyclodextrin significantly modifies the distribution of surfactants between the aqueous phase and the mixed micelles. Compared to the systems without βCD, the corrected micellar mole fractions become closer to the nominal surfactant composition, indicating reduced preferential incorporation and decreased selectivity of micellization. The differences between *x_i_* and *x_i_*(*K*) become more pronounced with increasing βCD concentration, suggesting that inclusion complex formation increasingly competes with micellization. This effect is particularly evident at 1 and 2 mM βCD, where substantial redistribution of surfactants occurs between the micellar and complexed states. In several systems, *x_i_*(*K*) exceeds *x_i_*, indicating preferential complexation of Triton X-100 monomers by β-cyclodextrin in the aqueous phase. As a consequence, the effective composition of the mixed micelles changes, reflecting the coupling between micellization and host–guest complexation equilibria.

The original RST protocol in the thermodynamic parameters of micellization induces an error due to the change in the $\alpha_{i}$ value in the binary mixture of surfactants due to the formation of an inclusion complex of surfactants with CD. While the modified RST model creates an error if the interaction parameter does not behave strictly according to RST, but has an entropy component, the size of which depends on the available space in the micellar particles.

The results presented in Table 5 and Table 6 indicate the formation of inclusion complexes between β-cyclodextrin and both surfactants (Triton X-100 and Brij S20). The equilibrium constant (*K*) values confirm that complexation occurs in all investigated systems, with values generally ranging from weak to moderate binding strength.

The calculated equilibrium constants further reveal differences between Triton X-100 and Brij S20. For Triton X-100, the equilibrium constants were generally moderate, whereas Brij S20 exhibited comparable or slightly lower values depending on temperature and β-CD concentration. These observations agree with literature reports indicating that Triton X-100 predominantly forms 1:1 inclusion complexes with β-CD through encapsulation of its hydrophobic aromatic moiety [32], whereas Brij surfactants interact more dynamically because β-CD can occupy different positions along the alkyl chain and the polyoxyethylene segment [5]. Nevertheless, other studies have also demonstrated the coexistence of 1:1 and 2:1 complexes for nonionic surfactants, particularly for polyoxyethylene alkyl ethers, depending on surfactant structure and experimental conditions [33,34].

### 3.3. Comparison of Inclusion Complex Formation in Triton X-100 and Brij S20 Systems

For Triton X-100 systems (Table 5), *K* values are in accordance with previously published results [36] and are typically higher than for Brij S20 (Table 6), suggesting stronger affinity of βCD toward Triton X-100. The higher equilibrium constants observed for Triton X-100 compared with Brij S20 can be rationalized by considering their structural differences. Triton X-100 contains a bulky aromatic octylphenyl group attached to a poly(ethylene oxide) chain, whereas Brij S20 consists of a linear stearyl chain linked to a poly(ethylene oxide) headgroup. The hydrophobic cavity of β-cyclodextrin is particularly well suited to accommodate aromatic and bulky hydrophobic moieties, allowing stronger van der Waals interactions and a better geometric fit than that achieved with a flexible linear alkyl chain. In addition, the phenyl ring of Triton X-100 is relatively rigid, which favors the formation of a more stable inclusion complex by reducing conformational flexibility upon binding. In contrast, the flexible stearyl chain of Brij S20 may adopt multiple conformations in solution, resulting in weaker and less specific host–guest interactions. Consequently, Triton X-100 generally exhibits higher inclusion equilibrium constants than Brij S20 under comparable experimental conditions.

Temperature plays an important role in determining the balance between surfactant micellization and β-cyclodextrin inclusion complex formation. In the absence of β-CD, the temperature dependence of the CMC values reflects the characteristic behavior of nonionic surfactants, where aggregation is governed by the balance between hydrophobic association and hydration of the polyoxyethylene groups. At lower temperatures, hydration of the ethoxylated chains is stronger, whereas increasing temperature promotes dehydration of the hydrophilic segments, which can facilitate micelle formation. However, further temperature increase may enhance molecular mobility and weaken the balance between hydrophobic interactions and head-group hydration, resulting in a more complex temperature dependence of micellization.

In the presence of β-CD, the experimentally determined CMC values are additionally affected by the simultaneous occurrence of surfactant inclusion and micellization equilibria. Increasing temperature modifies the extent of β-CD–surfactant complex formation by influencing hydrophobic interactions, cavity desolvation, and conformational changes in the surfactant molecules. Therefore, the observed temperature dependence of CMC values represents the combined contribution of surfactant aggregation and removal of surfactant monomers through β-CD complexation.

The temperature-dependent equilibrium constants indicate that β-cyclodextrin forms inclusion complexes with both Triton X-100 and Brij S20 throughout the investigated temperature range. The negative Gibbs free energy values obtained for all calculable systems confirm that complex formation is thermodynamically spontaneous.

For both surfactants, the calculated enthalpy changes are negative, demonstrating that inclusion complex formation is an exothermic process driven by favorable host–guest interactions within the β-cyclodextrin cavity. Such interactions most likely involve hydrophobic effects and van der Waals interactions between the hydrophobic portions of the surfactant molecules and the cyclodextrin cavity. Probably the flexible polyoxyethylene chains of the investigated surfactants, which are outside the hydrophobic cavity of cyclodextrin, form hydrogen bonds with the OH groups of cyclodextrin, which significantly increases the exothermicity of the formation of the inclusion complex.

The entropy contributions are comparatively small. For Triton X-100, slightly positive entropy values suggest that release of structured water molecules from the hydration shells of the guest and host molecules partially contributes to complex stabilization. In contrast, Brij S20 exhibits slightly negative entropy values, indicating a modest increase in system ordering upon inclusion, probably associated with restricted conformational freedom of the flexible alkyl chain within the β-cyclodextrin cavity. However, the magnitude of these entropy changes is small compared with the enthalpic contribution, indicating that complex formation in both systems is predominantly enthalpy-driven.

The calculated enthalpy and entropy changes exhibit only moderate variations with β-cyclodextrin concentration and temperature, suggesting that the thermodynamic mechanism of inclusion remains broadly similar throughout the investigated conditions.

Although slight variations in ΔH° and ΔS° are observed, the entropy contributions remain relatively small and do not substantially alter the predominantly enthalpy-driven nature of the inclusion process. Therefore, enthalpy–entropy compensation, although present to some extent, does not appear to be the dominant factor governing complex stability.

The thermodynamic analysis also supports previous calorimetric observations. Formation of both Triton X-100 and Brij S20 inclusion complexes is spontaneous, as demonstrated by the negative Gibbs free energy values. Moreover, the negative enthalpy changes indicate that complex formation is predominantly enthalpy-driven, consistent with earlier ITC studies on Brij homologues [5,35]. The comparatively small entropy contributions suggest that solvent reorganization and conformational changes accompany complex formation but do not represent the principal driving force of the inclusion process.

The formation of β-cyclodextrin inclusion complexes reduces the concentration of free surfactant molecules available for micellization, leading to increased *CMC* values and guest complexation, where increasing β-cyclodextrin concentration progressively shifts the equilibrium toward inclusion complex formation and away from micellization.

The thermodynamic analysis demonstrates that β-cyclodextrin forms spontaneous inclusion complexes with both Triton X-100 and Brij S20 throughout the investigated temperature range. Complex formation is predominantly enthalpy-driven, arising from favorable host–guest interactions within the β-cyclodextrin cavity, whereas entropy contributions are comparatively small. Triton X-100 generally exhibits higher equilibrium constants and more negative Gibbs free energy values than Brij S20 under comparable experimental conditions, indicating stronger host–guest affinity. These findings further support the view that β-cyclodextrin complexation effectively competes with surfactant micellization and significantly influences the aggregation behavior of nonionic surfactant systems.

Overall, while the obtained thermodynamic parameters are fully consistent with previous reports describing β-CD inclusion of nonionic surfactants, the present work extends existing knowledge by quantitatively describing how host–guest complexation modifies mixed micellization equilibria, mixed micelle composition, and surfactant–surfactant interactions in binary Triton X-100/Brij S20 systems.

## 4. Materials and Methods

### 4.1. Materials

β-Cyclodextrin (≥98%, Thermo Scientific, Shanghai, China), Brij S20 (Sigma-Aldrich, Darmstadt, Germany), Triton X-100 (Alfa Aesar, Karlsruhe, Germany), and pyrene (98%, Aldrich Chemistry, St. Louis, MI/Burlington, MA, USA) were used in this study.

### 4.2. Methods

#### 4.2.1. Spectrofluorimetric Measurements

The critical micelle concentrations (*CMC*) of the investigated surfactants were determined using spectrofluorimetric measurements. An Agilent Cary Eclipse fluorescence spectrophotometer (Agilent, Waldbronn, Germany), equipped with a Peltier temperature controller, was employed. Measurements were carried out over a temperature range of 278.15–318.15 K, with increments of 5 K.

The *CMC* values were determined using pyrene as a probe molecule. Pyrene was excited at a wavelength of 334 nm. The intensities of the first (I_1_, 373 nm) and third (I_3_, 384 nm) vibronic bands of the pyrene emission spectrum were monitored. Measurements were performed on a series of solutions with increasing total surfactant concentration. The ratio I_1_/I_3_ was plotted as a function of surfactant concentration, and the *CMC* values were obtained from these plots using Boltzmann fitting in OriginLab 9 software.

#### 4.2.2. Tensiometric Measurements

The *CMC* values of Brij S20 and Triton X-100 solutions, both in the absence and in the presence of β-cyclodextrin (*c*(β CD) = 0; 0.5, 1 and 2 mmol/dm^−3^), were confirmed by tensiometric measurements at 298.15 K. Measurements were performed using a Krüss K20 EasyDyne tensiometer (Krüss, Hamburg, Germany) applying the ring method.

## 5. Conclusions

The present study provides a comprehensive thermodynamic characterization of mixed micelles formed by Triton X-100 and Brij S20 in the absence and presence of β-cyclodextrin. The results confirm pronounced synergistic interactions between the two nonionic surfactants, as evidenced by negative interaction parameters, reduced critical micelle concentrations, and negative excess Gibbs free energies over the entire investigated temperature range.

The addition of β-cyclodextrin significantly modifies the micellization behavior of the mixed surfactant system. Increasing β-cyclodextrin concentration leads to higher CMC values, less negative interaction parameters, activity coefficients approaching unity, and reduced magnitudes of excess Gibbs free energy, indicating progressive weakening of surfactant–surfactant interactions. These effects arise from competitive inclusion complex formation between β-cyclodextrin and surfactant monomers, which decreases the concentration of free surfactant molecules available for micellization.

A major finding of this work is that inclusion complex formation alters the composition of the mixed micellar pseudophase and introduces deviations that cannot be fully explained by the classical regular solution theory. The obtained results indicate that the interaction parameter is influenced not only by intermolecular interaction energies but also by conformational contributions associated with changes in the available space within the micellar core. Consequently, conformational effects should be considered when interpreting interaction parameters in cyclodextrin–surfactant systems.

Thermodynamic analysis further demonstrated spontaneous formation of β-cyclodextrin inclusion complexes with both Triton X-100 and Brij S20. Triton X-100 exhibited stronger affinity toward β-cyclodextrin, whereas Brij S20 showed larger enthalpic and entropic changes upon complexation. The coupled equilibria between micellization and host–guest complex formation govern the overall behavior of the system and become increasingly important at higher β-cyclodextrin concentrations.

Overall, this work provides new insight into the interplay between mixed micellization and cyclodextrin complexation and highlights the need to account for conformational effects and coupled equilibria when describing cyclodextrin–surfactant systems. These findings contribute to a better understanding of supramolecular self-assembly processes relevant to pharmaceutical, cosmetic, and colloidal formulations.

## Figures and Tables

**Table 1:** Values of *CMC*, *CMCid*, *x_i_*, *x_j_*, *β_ij_*, *f_i_*, *f_j_* and *G^E^* for binary mixture of surfactants Triton X-100 and Brij S20, at various molar ratios of Triton X-100 over the temperature range *T* = (273.15–318.15) K, with 5 K increments calculated form the Regular Solution Theory.

*c*(βCD) = 0	*CMC*	*CMCid*	*x_i_*	*x_j_*	*β_ij_*	*f_i_*	*f_j_*	*G^E^*
	[mmol/dm^3^]							[kJ/mol]
*α*(TX100)/T[K]	273.15							
0	0.0253							
0.2	0.0181	0.0311	0.23	0.77	−5.27	0.05	0.75	−2.14
0.4	0.0221	0.0402	0.27	0.73	−4.45	0.09	0.72	−1.99
0.5	0.025	0.0471	0.29	0.71	−4.17	0.12	0.7	−1.96
0.6	0.0376	0.057	0.35	0.65	−3.96	0.19	0.91	−2.04
0.8	0.0456	0.0977	0.39	0.61	−3.48	0.27	0.59	−1.88
1	0.3437							
	278.15							
0	0.0221							
0.2	0.0223	0.0272	0.29	0.71	−6.07	0.05	1.14	−2.89
0.4	0.0194	0.0352	0.27	0.73	−4.51	0.09	0.72	−2.05
0.5	0.0189	0.0414	0.31	0.69	−4.95	0.09	0.62	−2.45
0.6	0.0318	0.0501	0.28	0.72	−2.95	0.21	0.8	−1.36
0.8	0.0387	0.0866	0.39	0.61	−3.71	0.25	0.57	−2.03
1	0.3209							
	283.15							
0	0.0307							
0.2	0.0215	0.0375	0.24	0.76	−5.12	0.05	0.74	−2.21
0.4	0.0253	0.0482	0.29	0.71	−4.43	0.11	0.69	−2.13
0.5	0.0239	0.0562	0.33	0.67	−4.96	0.11	0.58	−2.58
0.6	0.0242	0.0674	0.36	0.64	−5.27	0.12	0.5	−2.87
0.8	0.0469	0.1122	0.41	0.59	−3.80	0.27	0.52	−2.16
1	0.3338							
	288.15							
0	0.0425							
0.2	0.0291	0.0514	0.25	0.75	−4.75	0.07	0.73	−2.15
0.4	0.0221	0.0651	0.35	0.65	−6.00	0.08	0.48	−3.26
0.5	0.025	0.075	0.37	0.63	−5.63	0.11	0.47	−3.13
0.6	0.0445	0.0886	0.35	0.65	−3.46	0.24	0.65	−1.89
0.8	0.0492	0.1387	0.45	0.55	−4.28	0.27	0.42	−2.53
1	0.3195							
	293.15							
0	0.0538							
0.2	0.0326	0.0645	0.28	0.72	−5.01	0.07	0.67	−2.46
0.4	0.0425	0.0804	0.31	0.69	−3.72	0.17	0.69	−1.95
0.5	0.0325	0.0917	0.38	0.62	−5.09	0.14	0.48	−2.91
0.6	0.0394	0.1068	0.40	0.60	−4.56	0.19	0.49	−2.66
0.8	0.0675	0.1590	0.47	0.53	−3.47	0.37	0.47	−2.10
1	0.3109							
	298.15							
0	0.0565							
0.2	0.0332	0.0675	0.29	0.71	−5.08	0.08	0.66	−2.57
0.4	0.0425	0.0839	0.32	0.68	−3.86	0.17	0.67	−2.09
0.5	0.0312	0.0955	0.39	0.61	−5.38	0.13	0.45	−3.15
0.6	0.0454	0.1108	0.39	0.61	−4.07	0.22	0.53	−2.41
0.8	0.0831	0.1629	0.47	0.53	−2.72	0.46	0.55	−1.67
1	0.3078							
	303.15							
0	0.0425							
0.2	0.0435	0.0513	0.14	0.86	−2.15	0.21	0.96	−0.66
0.4	0.0565	0.0646	0.17	0.83	−1.18	0.45	0.97	−0.42
0.5	0.0473	0.0742	0.30	0.70	−2.64	0.27	0.79	−1.38
0.6	0.0490	0.0872	0.35	0.65	−2.90	0.29	0.71	−1.65
0.8	0.0771	0.1344	0.44	0.56	−2.30	0.48	0.64	−1.42
1	0.2923							
	308.15							
0	0.0512							
0.2	0.0456	0.0614	0.2	0.8	−2.93	0.15	0.89	−1.19
0.4	0.0514	0.0766	0.27	0.73	−2.59	0.25	0.83	−1.31
0.5	0.0584	0.0874	0.30	0.70	−2.29	0.33	0.81	−1.22
0.6	0.0512	0.1019	0.37	0.63	−3.26	0.28	0.64	−1.95
0.8	0.0842	0.1520	0.45	0.55	−2.25	0.50	0.60	−1.42
1	0.2994							
	313.15							
0	0.0684							
0.2	0.0471	0.0813	0.26	0.74	−4.13	0.11	0.75	−2.09
0.4	0.0625	0.1003	0.3	0.7	−2.8	0.25	0.78	−1.52
0.5	0.0608	0.1135	0.35	0.65	−3.16	0.26	0.68	−1.86
0.6	0.0612	0.1308	0.39	0.61	−3.44	0.28	0.59	−2.13
0.8	0.0905	0.1879	0.48	0.52	−2.94	0.45	0.51	−1.90
1	0.3338							
	318.15							
0	0.0711							
0.2	0.0546	0.0848	0.24	0.76	−3.65	0.12	0.81	−1.76
0.4	0.0743	0.1051	0.27	0.73	−2.23	0.3	0.85	−1.15
0.5	0.0718	0.1194	0.33	0.67	−2.69	0.3	0.75	−1.56
0.6	0.0728	0.1381	0.38	0.62	−2.98	0.31	0.66	−1.85
0.8	0.1045	0.2014	0.47	0.53	−2.64	0.48	0.56	−1.74
1	0.3717							

Standard uncertainties, *u*, are: *u*(*T*) = 0.1 K, *u*(*CMC*) = 0.005 mmol/dm^3^, *u*(*xi*) = 0.008, *u*(*xj*) = 0.008, *u*(*βij*) = 0.1, *u*(*fi*) = 0.006, *u*(*fj*) = 0.006, and *u*(*G^E^*) = 0.14 kJ mol^−1^.

**Table 2:** Values of *CMC*, *CMCid*, *x_i_*, *x_j_*, *β_ij_*, *f_i_*, *f_j_*, *x_i_*(*K*), *x_j_*(*K*) and *G^E^* for of mixed micelles composed of Triton X-100 and Brij S20 at various molar ratios (*α* denotes the mole fraction of Triton X-100) over the temperature range 273.15–318.15 K, with 5 K increments in the presence of 0.5 mmol/dm^3^ β-cyclodextrin.

	*CMC*	*CMCid*	*x_i_*	*x_j_*	*β_ij_*	*f_i_*	*f_j_*	*G^E^*	*x_i_*(*K*)	*x_j_*(*K*)
*c*(βCD) = 0.5 mM	[mmol/dm^3^]							[kJ/mol]		
*α*(TX100)/T[K]	273.15									
0	0.0339									
0.2	0.0383	0.0413	0.09	0.91	−1.57	0.27	0.99	−0.28	n.d.	n.d.
0.4	0.0462	0.0529	0.15	0.85	−1.43	0.36	0.97	−0.42	n.d.	n.d.
0.5	0.0555	0.0615	0.16	0.84	−0.94	0.52	0.98	−0.29	n.d.	n.d.
0.6	0.0725	0.0735	0.14	0.86	−0.12	0.92	1.00	−0.03	n.d.	n.d.
0.8	0.0934	0.1204	0.36	0.64	−1.15	0.62	0.86	−0.60	n.d.	n.d.
1	0.3327									
	278.15									
0	0.0479									
0.2	0.0428	0.0578	0.19	0.81	−3.16	0.13	0.89	−1.14	n.d.	n.d.
0.4	0.0571	0.0730	0.22	0.78	−1.92	0.31	0.91	−0.75	n.d.	n.d.
0.5	0.0755	0.0840	0.19	0.81	−0.81	0.59	0.97	−0.29	n.d.	n.d.
0.6	0.0985	0.0989	0.18	0.82	−0.03	0.98	1	−0.01	n.d.	n.d.
0.8	0.0971	0.1533	0.43	0.57	−2.01	0.53	0.72	−1.14	n.d.	n.d.
1	0.3409									
	283.15									
0	0.0452									
0.2	0.0515	0.0549	0.08	0.92	−1.32	0.33	0.99	−0.23	n.d.	n.d.
0.4	0.0585	0.0699	0.18	0.82	−1.64	0.33	0.95	−0.57	n.d.	n.d.
0.5	0.0781	0.0810	0.13	0.87	−0.35	0.77	0.99	−0.09	n.d.	n.d.
0.6	0.0908	0.0962	0.19	0.82	−0.41	0.76	0.99	−0.15	n.d.	n.d.
0.8	0.0813	0.1542	0.42	0.58	−2.72	0.40	0.62	−1.56	n.d.	n.d.
1	0.3879									
	288.15									
0	0.0512									
0.2	0.0525	0.0623	0.14	0.86	−2.47	0.16	0.95	−0.71	0.21	0.78
0.4	0.0606	0.0795	0.21	0.79	−2.28	0.24	0.90	−0.91	0.33	0.67
0.5	0.0697	0.0923	0.24	0.76	−2.00	0.31	0.89	−0.87	0.33	0.66
0.6	0.0975	0.1100	0.21	0.79	−0.83	0.60	0.96	−0.33	0.31	0.68
0.8	0.094	0.1781	0.41	0.59	−2.74	0.39	0.63	−1.59	0.43	0.56
1	0.4679									
	293.15									
0	0.0538									
0.2	0.0422	0.0655	0.22	0.78	−4.29	0.07	0.81	−1.81	n.d.	n.d.
0.4	0.0606	0.0837	0.23	0.77	−2.6	0.21	0.87	−1.11	n.d.	n.d.
0.5	0.0734	0.0972	0.24	0.76	−2.02	0.31	0.89	−0.89	n.d.	n.d.
0.6	0.103	0.1160	0.21	0.79	−0.83	0.59	0.97	−0.33	n.d.	n.d.
0.8	0.1078	0.1886	0.40	0.60	−2.43	0.42	0.67	−1.42	n.d.	n.d.
1	0.5051									
	298.15									
0	0.0538									
0.2	0.0456	0.0657	0.20	0.80	−3.99	0.08	0.85	−1.59	n.d.	n.d.
0.4	0.0559	0.0844	0.24	0.76	−3.21	0.16	0.83	−1.47	n.d.	n.d.
0.5	0.0720	0.0984	0.24	0.76	−2.35	0.26	0.88	−1.07	n.d.	n.d.
0.6	0.1054	0.1179	0.19	0.81	−0.84	0.58	0.97	−0.32	n.d.	n.d.
0.8	0.1274	0.1955	0.38	0.62	−1.93	0.47	0.76	−1.12	n.d.	n.d.
1	0.5728									
	303.15									
0	0.0565									
0.2	0.0594	0.0693	0.12	0.88	−2.64	0.13	0.96	−0.72	0.09	0.90
0.4	0.0675	0.0895	0.20	0.8	−2.65	0.18	0.90	−1.08	0.13	0.86
0.5	0.0962	0.1048	0.13	0.87	−0.94	0.49	0.98	−0.27	0.19	0.81
0.6	0.1043	0.1265	0.21	0.79	−1.45	0.41	0.94	−0.61	0.24	0.76
0.8	0.1205	0.2153	0.38	0.62	−2.68	0.35	0.68	−1.58	0.38	0.62
1	0.7243									
	308.15									
0	0.0565									
0.2	0.0503	0.0695	0.18	0.82	−4.12	0.06	0.87	−1.58	0.16	0.84
0.4	0.0793	0.0902	0.13	0.87	−1.7	0.28	0.97	−0.50	0.13	0.87
0.5	0.0848	0.1061	0.19	0.81	−2.07	0.26	0.93	−0.82	0.18	0.81
0.6	0.1205	0.1286	0.14	0.86	−0.65	0.61	0.99	−0.20	0.20	0.80
0.8	0.1216	0.2239	0.37	0.63	−2.92	0.31	0.68	−1.73	0.38	0.61
1	0.8637									
	313.15									
0	0.0598									
0.2	0.0607	0.0735	0.14	0.86	−3.10	0.10	0.94	−0.96	n.d.	n.d.
0.4	0.0741	0.0953	0.19	0.81	−2.58	0.18	0.91	−1.02	n.d.	n.d.
0.5	0.1078	0.1119	0.14	0.86	−1.09	0.45	0.92	−0.34	n.d.	n.d.
0.6	0.1134	0.1356	0.20	0.80	−1.44	0.39	0.95	−0.60	n.d.	n.d.
0.8	0.1261	0.2347	0.37	0.63	−2.93	0.31	0.67	−1.78	n.d.	n.d.
1	0.8733									
	318.15									
0	0.0651									
0.2	0.0663	0.08	0.14	0.86	−3.05	0.10	0.94	−0.95	n.d.	n.d.
0.4	0.0882	0.1037	0.15	0.85	−1.92	0.25	0.96	−0.65	n.d.	n.d.
0.5	0.1078	0.1217	0.15	0.85	−1.3	0.39	0.97	−0.43	n.d.	n.d.
0.6	0.1284	0.1473	0.18	0.82	−1.17	0.46	0.96	−0.46	n.d.	n.d.
0.8	0.1467	0.2545	0.36	0.64	−2.62	0.35	0.71	−1.60	n.d.	n.d.
1	0.9339									

Standard uncertainties, *u*, are: *u*(*T*) = 0.1 K, *u*(*CMC*) = 0.006 mmol/dm^3^, *u*(*xi*) = 0.008, *u*(*xj*) = 0.008, *u*(*βij*) = 0.1, *u*(*fi*) = 0.05, *u*(*fj*) = 0.05, *u*(*G^E^*) = 0.14 kJ mol^−1^, *u*(*xi*(*K*)) = 0.005 and *u*(*xj*(*K*)) = 0.005.

**Table 3:** Values of *CMC*, *CMCid*, *x_i_*, *x_j_*, *β_ij_*, *f_i_*, *f_j_*, *x_i_*(*K*), *x_j_*(*K*) and *G^E^* for of mixed micelles composed of Triton X-100 and Brij S20 at various molar ratios (*α* denotes the mole fraction of Triton X-100) over the temperature range 273.15–318.15 K, with 5 K increments in the presence of 1 mmol/dm^3^ β-cyclodextrine.

	*CMC*	*CMCid*	*x_i_*	*x_j_*	*β_ij_*	*f_i_*	*f_j_*	G^E^	*x_i_*(*K*)	*x_j_*(*K*)
*c*(βCD) = 1 mM	[mmol/dm^3^]							[kJ/mol]		
*α*(TX100) /T[K]	273.15									
0	0.0829									
0.2	0.0844	0.1009	0.14	0.86	−2.54	0.15	0.95	−0.70	0.23	0.77
0.4	0.0844	0.1310	0.25	0.75	−3.14	0.17	0.82	−1.35	0.26	0.74
0.5	0.1119	0.1521	0.24	0.76	−2.06	0.30	0.89	−0.85	0.29	0.71
0.6	0.1119	0.2295	0.31	0.69	−2.62	0.28	0.78	−1.27	0.33	0.67
0.8	0.0916	0.3532	0.44	0.56	−4.84	0.22	0.39	−2.71	0.39	0.61
1	0.7665									
	278.15									
0	0.0856									
0.2	0.0981	0.1045	0.08	0.92	−1.45	0.29	0.99	−0.23	0.24	0.76
0.4	0.1188	0.1524	0.14	0.86	−1.36	0.37	0.97	−0.39	0.25	0.75
0.5	0.1242	0.2098	0.21	0.79	−1.81	0.33	0.92	−0.70	0.29	0.71
0.6	0.1257	0.2570	0.29	0.71	−2.4	0.29	0.82	−1.13	0.26	0.74
0.8	0.1031	0.4025	0.43	0.57	−4.71	0.21	0.42	−2.67	0.37	0.63
1	0.8956									
	283.15									
0	0.0829									
0.2	0.0876	0.1013	0.12	0.88	−2.42	0.16	0.96	−0.62	0.20	0.80
0.4	0.1104	0.1303	0.16	0.84	−1.74	0.30	0.95	−0.56	0.24	0.76
0.5	0.1327	0.1520	0.17	0.83	−1.24	0.43	0.96	−0.41	0.28	0.72
0.6	0.1265	0.1825	0.27	0.73	−2.28	0.30	0.84	−1.07	0.32	0.68
0.8	0.1239	0.3043	0.41	0.59	−3.92	0.26	0.51	−2.24	0.37	0.63
1	0.9161									
	288.15									
0	0.0883									
0.2	0.0753	0.1546	0.20	0.80	−3.76	0.09	0.86	−1.46	0.21	0.79
0.4	0.1098	0.1937	0.20	0.80	−1.97	0.28	0.93	−0.74	0.3	0.7
0.5	0.1304	0.2217	0.21	0.79	−1.53	0.39	0.93	−0.61	0.32	0.68
0.6	0.1201	0.2593	0.31	0.69	−2.56	0.29	0.79	−1.31	0.30	0.70
0.8	0.1308	0.3921	0.43	0.57	−3.62	0.30	0.52	−2.12	0.40	0.60
1	0.8037									
	293.15									
0	0.0651									
0.2	0.0706	0.1415	0.11	0.89	−2.53	0.13	0.97	−0.59	0.14	0.86
0.4	0.0891	0.1801	0.15	0.86	−1.91	0.25	0.96	−0.58	0.16	0.84
0.5	0.1188	0.2086	0.08	0.92	−0.42	0.70	1.00	−0.08	0.20	0.80
0.6	0.1204	0.2478	0.21	0.79	−1.66	0.35	0.93	−0.66	0.22	0.78
0.8	0.1583	0.3969	0.35	0.65	−2.39	0.36	0.75	−1.32	0.31	0.69
1	0.9962									
	298.15									
0	0.0625									
0.2	0.0753	0.0769	0.04	0.96	−0.92	0.42	1.00	−0.08	0.12	0.88
0.4	0.096	0.1000	0.07	0.93	−0.78	0.51	1.00	−0.13	0.13	0.87
0.5	0.1135	0.1177	0.09	0.91	−0.54	0.64	1.00	−0.11	0.18	0.82
0.6	0.1255	0.1430	0.17	0.83	−1.19	0.44	0.97	−0.41	0.19	0.81
0.8	0.1342	0.2505	0.36	0.64	−3.02	0.29	0.67	−1.73	0.35	0.65
1	1.0107									
	303.15									
0	0.0698									
0.2	0.0794	0.0858	0.08	0.92	−1.9	0.20	0.99	−0.35	0.05	0.95
0.4	0.105	0.1112	0.09	0.91	−0.93	0.46	0.99	−0.19	0.06	0.94
0.5	0.1214	0.1305	0.12	0.88	−0.88	0.51	0.99	−0.23	0.14	0.86
0.6	0.1257	0.1580	0.22	0.78	−1.74	0.34	0.92	−0.75	0.19	0.81
0.8	0.1388	0.2731	0.38	0.62	−3.16	0.29	0.64	−1.87	0.32	0.68
1	1.0048									
	308.15									
0	0.0615									
0.2	0.0722	0.0756	0.06	0.94	−1.40	0.29	1.00	−0.19	0.17	0.83
0.4	0.095	0.0980	0.11	0.89	−1.13	0.41	1.04	−0.27	0.23	0.77
0.5	0.1295	0.1151	0.13	0.87	−0.79	0.55	1.21	−0.23	0.25	0.75
0.6	0.1352	0.1393	0.12	0.88	−0.32	0.78	1.00	−0.08	0.32	0.68
0.8	0.1605	0.2409	0.34	0.66	−1.99	0.42	0.79	−1.15	0.41	0.59
1	0.8902									
	313.15									
0	0.0725									
0.2	0.0766	0.0888	0.12	0.88	−2.54	0.14	0.96	−0.71	0.13	0.87
0.4	0.0981	0.1146	0.15	0.85	−1.75	0.29	0.96	−0.59	0.15	0.85
0.5	0.1326	0.1341	0.11	0.89	−0.46	0.69	1.02	−0.11	0.14	0.86
0.6	0.1489	0.1615	0.16	0.84	−0.69	0.61	0.98	−0.25	0.19	0.81
0.8	0.2422	0.2734	0.30	0.70	−0.61	0.74	0.95	−0.33	0.26	0.74
1	0.8902									
	318.15									
0	0.0781									
0.2	0.0775	0.0969	0.15	0.85	−3.13	0.10	0.93	−1.05	0.14	0.86
0.4	0.1029	0.1285	0.17	0.83	−2.00	0.25	0.95	−0.73	0.14	0.86
0.5	0.1358	0.1530	0.19	0.81	−1.54	0.36	0.81	−0.62	0.14	0.86
0.6	0.1512	0.1897	0.19	0.81	−1.15	0.47	0.55	−0.47	0.19	0.81
0.8	0.2502	0.3559	0.30	0.70	−0.88	0.65	0.48	−0.49	0.27	0.73
1	1.0048									

Standard uncertainties, *u*, are: *u*(*T*) = 0.1 K, *u*(*CMC*) = 0.006 mmol dm^−3^, *u*(*xi*) = 0.008, *u*(*xj*) = 0.008, *u*(*βij*) = 0.1, *u*(*fi*) = 0.05, *u*(*fj*) = 0.05, *u*(*G^E^*) = 0.14 kJ mol^−1^, *u*(*xi*(*K*)) = 0.005 and *u*(*xj*(*K*)) = 0.005.

**Table 4:** Values of *CMC*, *CMCid*, *x_i_*, *x_j_*, *β_ij_*, *f_i_*, *f_j_*, *x_i_*(*K*), *x_j_*(*K*) and *G^E^* for of mixed micelles composed of Triton X-100 and Brij S20 at various molar ratios (*α* denotes the mole fraction of Triton X-100) over the temperature range 273.15–318.15 K, with 5 K increments in the presence of 2 mmol/dm^3^ β-cyclodextrine.

	*CMC*	*CMCid*	*x_i_*	*x_j_*	*β_ij_*	*f_i_*	*f_j_*	*G^e^*	*x_i_*(*K*)	*x_j_*(*K*)
*c*(βCD) = 2 mM	[mmol/dm^3^]							[kJ/mol]		
*α*(TX100) /T[K]		273.15								
0	0.131									
0.2	0.0925	0.1608	0.24	0.76	−5.37	0.04	0.74	−2.20	0.24	0.76
0.4	0.1025	0.2082	0.29	0.71	−5.00	0.08	0.66	−2.33	0.28	0.72
0.5	0.1214	0.2441	0.30	0.70	−4.49	0.11	0.66	−2.14	0.3	0.7
0.6	0.1431	0.2951	0.32	0.68	−4.17	0.15	0.65	−2.07	0.33	0.67
0.8	0.2265	0.5066	0.39	0.61	−3.67	0.26	0.57	−1.98	0.40	0.60
1	1.7884									
	278.15									
0	0.1284									
0.2	0.1028	0.1578	0.21	0.79	−4.73	0.05	0.81	−1.81	0.26	0.74
0.4	0.1254	0.2046	0.25	0.75	−3.97	0.11	0.78	−1.71	0.28	0.72
0.5	0.1286	0.2403	0.29	0.71	−4.21	0.12	0.70	−1.99	0.32	0.68
0.6	0.1743	0.2911	0.29	0.71	−3.25	0.19	0.76	−1.54	0.29	0.71
0.8	0.2609	0.5038	0.37	0.63	−3.09	0.30	0.65	−1.67	0.40	0.60
1	1.8727									
	283.15									
0	0.1305									
0.2	0.1166	0.1435	0.18	0.82	−4.02	0.07	0.87	−1.41	0.23	0.77
0.4	0.1431	0.1866	0.22	0.78	−3.3	0.14	0.85	−1.34	0.28	0.73
0.5	0.1689	0.2195	0.24	0.76	−2.87	0.19	0.85	−1.22	0.32	0.68
0.6	0.2233	0.2666	0.23	0.77	−2.02	0.31	0.89	−0.85	0.35	0.65
0.8	0.2636	0.4666	0.38	0.62	−3.08	0.30	0.65	−1.70	0.40	0.60
1	1.8689									
	288.15									
0	0.1286									
0.2	0.1395	0.1712	0.11	0.89	−2.49	0.14	0.97	−0.57	0.21	0.79
0.4	0.1689	0.2216	0.16	0.84	−2.19	0.22	0.94	−0.71	0.31	0.69
0.5	0.1977	0.2599	0.18	0.82	−1.87	0.29	0.94	−0.66	0.32	0.68
0.6	0.2867	0.3141	0.11	0.89	−0.20	0.85	1.00	−0.044	0.29	0.71
0.8	0.2749	0.5390	0.37	0.63	−2.89	0.32	0.68	−1.61	0.40	0.60
1	1.8968									
	293.15									
0	0.1165									
0.2	0.1402	0.1720	0.12	0.88	−2.73	0.12	1.10	−0.71	0.21	0.79
0.4	0.1782	0.2225	0.22	0.78	−1.68	0.36	1.17	−0.69	0.22	0.78
0.5	0.2376	0.2607	0.20	0.8	−1.46	0.40	1.28	−0.57	0.29	0.71
0.6	0.2408	0.3148	0.21	0.79	−1.94	0.30	1.05	−0.78	0.31	0.69
0.8	0.3166	0.5382	0.25	0.76	−1.46	0.43	0.72	−0.65	0.37	0.63
1	1.8542									
	298.15									
0	0.0929									
0.2	0.1489	0.1149	0.13	0.87	−2.95	0.11	1.47	−0.82	0.16	0.84
0.4	0.2265	0.1505	0.15	0.85	−1.83	0.27	1.73	−0.59	0.17	0.83
0.5	0.2378	0.1782	0.17	0.83	−1.59	0.33	1.53	−0.54	0.25	0.75
0.6	0.3067	0.2183	0.18	0.82	−1.15	0.46	1.61	−0.42	0.24	0.76
0.8	0.3185	0.3968	0.25	0.75	−1.37	0.46	0.92	−0.64	0.29	0.71
1	2.1765									
	303.15									
0	0.0969									
0.2	0.1558	0.1196	0.24	0.76	−3.46	0.13	1.69	−1.58	0.07	0.93
0.4	0.2206	0.1563	0.23	0.77	−1.58	0.39	1.78	−0.70	0.08	0.92
0.5	0.2233	0.1845	0.24	0.76	−1.3	0.47	1.52	−0.60	0.19	0.81
0.6	0.2895	0.2253	0.26	0.74	−0.69	0.69	1.62	−0.33	0.23	0.77
0.8	0.3841	0.4035	0.33	0.67	−0.08	0.96	1.18	−0.04	0.31	0.69
1	1.9319									
	308.15									
0	0.0969									
0.2	0.1188	0.1196	0.17	0.83	−2.72	0.15	1.18	−0.96	0.11	0.89
0.4	0.1921	0.1562	0.21	0.79	−1.53	0.39	1.51	−0.65	0.15	0.85
0.5	0.2265	0.1844	0.23	0.77	−1.06	0.53	1.51	−0.47	0.16	0.84
0.6	0.3271	0.225	0.14	0.86	−1.64	0.3	1.57	−0.50	0.23	0.77
0.8	0.5078	0.4024	0.21	0.79	−1.40	0.42	1.32	−0.59	0.29	0.71
1	1.8987									
	313.15									
0	0.0942									
0.2	0.1395	0.1162	0.16	0.84	−3.35	0.1	1.41	−1.18	0.12	0.88
0.4	0.1948	0.1517	0.19	0.81	−2.29	0.22	1.54	−0.92	0.12	0.88
0.5	0.2319	0.1791	0.21	0.79	−1.87	0.31	1.55	−0.80	0.16	0.84
0.6	0.2895	0.2184	0.22	0.78	−1.32	0.44	1.57	−0.58	0.2	0.8
0.8	0.482	0.3897	0.32	0.68	−0.86	0.67	1.50	−0.48	0.26	0.74
1	1.8049									
	318.15									
0	0.1172									
0.2	0.1326	0.1441	0.17	0.83	−3.58	0.09	1.09	−1.35	0.15	0.85
0.4	0.1902	0.1872	0.20	0.8	−2.44	0.21	1.22	−1.03	0.15	0.85
0.5	0.2351	0.2200	0.21	0.79	−1.87	0.31	1.27	−0.82	0.20	0.80
0.6	0.2695	0.2669	0.23	0.77	−1.64	0.38	1.20	−0.77	0.25	0.75
0.8	0.4648	0.4647	0.38	0.62	−1.53	0.55	1.27	−0.94	0.32	0.68
1	1.7967									

Standard uncertainties, *u*, are: *u*(*T*) = 0.1 K, *u*(*CMC*) = 0.006 mmol/dm^3^, *u*(*xi*) = 0.008, *u*(*xj*) = 0.008, *u*(*βij*) = 0.1, *u*(*fi*) = 0.05, *u*(*fj*) = 0.05, *u*(*G^E^*) = 0.14 kJ mol^−1^, *u*(*xi*(*K*)) = 0.005 and *u*(*xj*(*K*)) = 0.005.

**Table 5:** Values of the calculated standard equilibrium constants for the formation of inclusion complexes between Triton X-100 and different concentrations of β-cyclodextrin (0, 0.5, 1, and 2 mmol/dm^3^), denoted as *K*[CD–S], as well as the calculated Gibbs free energy changes, Δ*G*, the enthalpy Δ*H* and entropy Δ*S* changes.

$C_{CD}^{T}$ **/T[K]**	CMC(S)	$CMC\left(CD\right)$	$CMC(S)∙C_{CD}^{T}$	∆CMC	*K* ^0^ *·10^−^* ^3^	Δ*G*^0^	Δ*H*^0^	Δ*S*^0^
mmol/dm^3^						[kJ/mol]	[kJ/mol]	[J/mol·K]
273.15								
0	0.3440		0	0				
0.5	0.3440	0.33	0.17	−0.01	n.d.	n.d.	n.d.	n.d.
1	0.3440	0.77	0.34	0.42	2.13	−17.41	−17.43	0.09
2	0.3440	1.79	0.69	1.44	7.57	−20.28	−20.30	0.05
278.15								
0	0.3210		0	0				
0.5	0.3210	0.34	0.16	0.02	n.d.	n.d.	n.d.	n.d.
1	0.3210	0.90	0.32	0.57	4.21	−19.30	−19.32	0.09
2	0.3210	1.87	0.64	1.55	10.79	−21.47	−21.49	0.05
283.15								
0	0.3340		0	0				
0.5	0.3340	0.39	0.17	0.05	0.36	−13.88	−14.02	0.51
1	0.3340	0.92	0.33	0.58	4.18	−19.63	−19.65	0.09
2	0.3340	1.87	0.67	1.54	9.89	−21.66	−21.67	0.05
288.15								
0	0.3200		0	0				
0.5	0.3200	0.47	0.16	0.15	1.32	−17.22	−17.36	0.51
1	0.3200	0.80	0.32	0.48	2.94	−19.13	−19.16	0.09
2	0.3200	1.90	0.64	1.58	11.68	−22.44	−22.45	0.05
293.15								
0	0.3110		0	0				
0.5	0.3110	0.51	0.16	0.19	2.04	−18.58	−18.73	0.51
1	0.3110	1.00	0.31	0.69	7.00	−21.58	−21.61	0.09
2	0.3110	1.85	0.62	1.54	10.87	−22.65	−22.67	0.05
298.15								
0	0.3080		0	0				
0.5	0.3080	0.57	0.15	0.27	3.66	−20.34	−20.50	0.51
1	0.3080	1.01	0.31	0.70	7.69	−22.18	−22.21	0.09
2	0.3080	2.18	0.62	1.87	46.24	−26.63	−26.64	0.05
303.15								
0	0.2920		0	0				
0.5	0.2920	0.72	0.15	0.43	21.73	−25.17	−25.33	0.51
1	0.2920	1.00	0.29	0.71	8.48	−22.80	−22.83	0.09
2	0.2920	1.93	0.58	1.64	15.56	−24.33	−24.34	0.05
308.15								
0	0.2990		0	0				
0.5	0.2990	0.86	0.15	0.56	n.d.	n.d.	n.d.	n.d.
1	0.2990	0.89	0.30	0.59	4.82	−21.73	−21.76	0.09
2	0.2990	1.90	0.60	1.60	13.33	−24.33	−24.35	0.05
313.15								
0	0.3340		0	0				
0.5	0.3340	0.87	0.17	0.54	n.d.	n.d.	n.d.	n.d.
1	0.3340	0.89	0.33	0.56	3.76	−21.43	−21.46	0.09
2	0.3340	1.80	0.67	1.47	8.33	−23.50	−23.52	0.05
318.15								
0	0.3720		0	0				
0.5	0.3720	0.93	0.19	0.56	n.d.	n.d.	n.d.	n.d.
1	0.3720	1.00	0.37	0.63	4.64	−22.33	−22.36	0.09
2	0.3720	1.80	0.74	1.43	6.67	−23.29	−23.31	0.05

Standard uncertainties, *u*, are: *u*(*T*) = 0.1 K, *u*(*CMC*) = 0.005 mmol dm^−3^, *u*(*K*) = 0.01, *u*(Δ*G*) = 0.08 kJ mol^−1^, *u*(Δ*H*) = 0.08 kJ mol^−1^, *u*(Δ*S*) = 0.01 J K^−1^ mol^−1^.

**Table 6:** Values of the calculated standard equilibrium constants for the formation of inclusion complexes between Brij S20 and different concentrations of β-cyclodextrin (0, 0.5, 1, and 2 mmol/dm^3^), denoted as *K*[CD–S], as well as the calculated Gibbs free energy changes, Δ*G*, the enthalpy Δ*H* and entropy Δ*S* changes.

$C_{CD}^{T}$ **/T[K]**	CMC(S)	$CMC\left(CD\right)$	$CMC(S)∙C_{CD}^{T}$	∆CMC	*K* ^0^ *·10* ^3^	ΔG^0^	Δ*H*^0^	ΔS^0^
mmol/dm^3^						[kJ/mol]	[kJ/mol]	[J/mol·K]
273.15								
0	0.0253		0	0				
0.5	0.0253	0.03	0.01	0.01	0.69	−14.85	−14.89	−0.13
1	0.0253	0.08	0.03	0.06	2.42	−17.69	−17.73	−0.16
2	0.0253	0.13	0.05	0.11	2.21	−17.48	−17.50	−0.06
278.15								
0	0.0221		0	0				
0.5	0.0221	0.05	0.01	0.03	2.46	−18.06	−18.09	−0.13
1	0.0221	0.09	0.02	0.06	3.07	−18.57	−18.61	−0.16
2	0.0221	0.13	0.04	0.11	2.54	−18.13	−18.15	−0.06
283.15								
0	0.0307		0	0				
0.5	0.0307	0.05	0.02	0.01	0.97	−16.20	−16.23	−0.13
1	0.0307	0.08	0.03	0.05	1.79	−17.64	−17.68	−0.16
2	0.0307	0.13	0.06	0.1	1.71	−17.53	−17.54	−0.06
288.15								
0	0.0425		0	0				
0.5	0.0425	0.05	0.02	0.01	0.42	−14.45	−14.49	−0.13
1	0.0425	0.09	0.04	0.05	1.13	−16.84	−16.89	−0.16
2	0.0425	0.13	0.09	0.09	1.06	−16.68	−16.70	−0.06
293.15								
0	0.0538		0	0				
0.5	0.0538	0.05	0.03	0	n.d.	n.d.	n.d.	n.d.
1	0.0538	0.07	0.05	0.01	0.21	−13.06	−13.11	−0.16
2	0.0538	0.12	0.11	0.06	0.60	−15.60	−15.61	−0.06
298.15								
0	0.0565		0	0				
0.5	0.0565	0.05	0.03	0.00	n.d.	n.d.	n.d.	n.d
1	0.0565	0.06	0.06	0.01	0.11	−11.58	−11.63	−0.16
2	0.0565	0.09	0.11	0.04	0.33	−14.36	−14.38	−0.06
303.15								
0	0.0425		0	0				
0.5	0.0425	0.06	0.02	0.01	0.68	−16.43	−16.47	−0.13
1	0.0425	0.07	0.04	0.03	0.66	−16.36	−16.41	−0.16
2	0.0425	0.10	0.09	0.05	0.66	−16.35	−16.37	−0.06
308.15								
0	0.0512		0	0				
0.5	0.0512	0.06	0.03	0.01	0.21	−13.69	−13.73	−0.13
1	0.0512	0.06	0.05	0.01	0.20	−13.62	−13.67	−0.16
2	0.0512	0.10	0.10	0.05	0.46	−15.69	−15.71	−0.06
313.15								
0	0.0684		0	0				
0.5	0.0684	0.06	0.03	n.d.	n.d.	n.d.	n.d.	n.d.
1	0.0684	0.07	0.07	0	0.06	−10.67	−10.72	−0.16
2	0.0684	0.09	0.14	0.03	0.19	−13.68	−13.69	−0.06
318.15								
0	0.0711		0					
0.5	0.0711	0.07	0.04	n.d.	n.d.	n.d.	n.d.	n.d.
1	0.0711	0.08	0.07	0.01	0.10	−12.16	−12.21	−0.16
2	0.0711	0.12	0.14	0.05	0.33	−15.35	−15.37	−0.06

Standard uncertainties, *u*, are: *u*(*T*) = 0.1 K, *u*(*CMC*) = 0.005 mmol/dm^3^, *u*(*K*) = 0.01, *u*(Δ*G*) = 0.08 kJ mol^−1^, *u*(Δ*H*) = 0.08 kJ mol^−1^, *u*(Δ*S*) = 0.01 J K^−1^ mol^−1^.

## Data Availability

Our data is unavailable due to privacy reasons.

## Abbreviations

The following abbreviations are used in this manuscript:

CD	cyclodextrin
*CMC*	critical micelle concentration
BS20	Brij S20
PEO	polyoxyethylene
OE	oxyethylene
TX100	Triton X-100
RST	regular solution theory

## Appendix A

In Table A1 are presented tensiometric measurments of critical micelle concentrations of pure surfactants Triton X-100 and Brij S20 (in relation 1:1), and in the presence of 0.5 mmol/dm^3^, 1 mmol/dm^3^ and 2 mmol/dm^3^ β-cyclodextrine on 298.15 K.

**Table A1:** Values of critical micelle concentrations of pure surfactants Triton X-100 and Brij S20 (in relation 1:1), and in the presence of 0.5 mmol/ dm^3^, 1 mmol/ dm^3^ and 2 mmol/dm^3^ β-cyclodextrine on 298.15 K obtained by tensiometric measurment.

	*CMC*			
*α* (TX100)/T[K]	*c*(βCD) = 0	*c*(βCD) = 0.5	*c*(βCD) = 1	*c*(βCD) = 2
	[mmol/dm^3^]			
0	0.053	0.0585	0.0624	0.079
0.5	0.0295	0.0682	0.1235	0.1889
1	0.272	0.5852	1.0018	2.2141
